# *A bit of medical paternalism?* A qualitative study on power relations between women and healthcare providers when deciding on mode of birth in five public maternity wards of Argentina

**DOI:** 10.1186/s12978-023-01661-5

**Published:** 2023-08-21

**Authors:** M. Vila Ortiz, C. Gialdini, C. Hanson, A. P. Betrán, G. Carroli, H. Mølsted Alvesson

**Affiliations:** 1 Department of Global Public Health, Karolinska Institutet, Stockholm, Sweden; 2 Facultad de Ciencias de la Salud Blanquerna, Universidad Ramón Llull, Barcelona, Spain; 3 Centro Rosarino de Estudios Perinatales, Rosario, Argentina; 4 UNDP/UNFPA/UNICEF/WHO/World Bank Special Programme of Research, Development and Research Training in Human Reproduction, Department of Sexual and Reproductive Health and Research, World Health Organization, Geneva, Switzerland

**Keywords:** Mode of birth, Decision-making, Caesarean section, Power relations, Medical paternalism

## Abstract

**Background:**

Whether women should be able to decide on mode of birth in healthcare settings has been a topic of debate in the last few decades. In the context of a marked increase in global caesarean section rates, a central dilemma is whether pregnant women should be able to request this procedure without medical indication. Since 2015, Law 25,929 of Humanised Birth is in place in Argentina. This study aims at understanding the power relations between healthcare providers, pregnant women, and labour companions regarding decision-making on mode of birth in this new legal context. To do so, central concepts of power theory are used.

**Methods:**

This study uses a qualitative design. Twenty-six semi-structured interviews with healthcare providers were conducted in five maternity wards in different regions of Argentina. Participants were purposively selected using heterogeneity sampling and included obstetrician/gynaecologists (heads of department, specialists working in 24-h shifts, and residents) and midwives where available. Reflexive thematic analysis was used to inductively develop themes and categories.

**Results:**

Three themes were developed: (1) Healthcare providers reconceptualize decision-making processes of mode of birth to make women’s voices matter; (2) Healthcare providers feel powerless against women’s request to choose mode of birth; (3) Healthcare providers struggle to redirect women’s decision regarding mode of birth. An overarching theme was built to explain the power relations between healthcare providers, women and labour companions: Healthcare providers’ loss of *beneficial power* in decision-making on mode of birth.

**Conclusions:**

Our analysis highlights the complexity of the healthcare provider-woman interaction in a context in which women are, in practice, allowed to choose mode of birth. Even though healthcare providers claim to welcome women being an active part of the decision-making processes, they feel powerless when women make autonomous decisions regarding mode of birth. They perceive themselves to be losing *beneficial power* in the eyes of patients and consider fruitful communication on risks and benefits of each mode of birth to not always be possible. At the same time, providers perform an increasing number of CSs without medical indication when it is convenient for them, which suggests that paternalistic practices are still in place.

## Plain Language Summary

In the last few decades, there has been a debate on whether women should be able to choose if they haver a vaginal birth or a caesarean section. This debate has been framed by the fact that an increasing number of caesarean sections are being performed. Since 2015, Argentina has a Law of Humanised Birth. We conducted a study to understand the power relations between healthcare providers, pregnant women and labour companions in decision making on mode of birth in this new legal context. To do so, we used central concepts of power theory. We conducted 26 semi-structured interviews with healthcare providers in five maternity wards of Argentina. The interviewees were obstetrician/gynaecologists (heads of department, specialists working in 24-h shifts, and residents) and midwives where available. We used thematic analysis to build themes from the data. We discovered that healthcare providers perceive themselves to be losing *beneficial power* in decision-making on mode of birth. Even though they claim to want women to make autonomous decisions, they feel frustrated when this happens. They also perceive it to be more difficult to communicate with patients regarding the risks and benefits of vaginal birth and caesarean section. At the same time, providers carry out an increasing number of CSs without medical indication when it is convenient for them, which suggests that paternalistic practices are still in place.

## Background

Women being able to decide on mode of birth in healthcare settings has been a topic of debate in the last few decades, giving rise to arguments both in favour and against this practice [1–9]. Self-determination movements have emphasised women’s right to make decisions regarding their health, supporting their reasoning with the principle of patient autonomy [2, 4, 5, 10, 11]. Those who argue against giving women the right to choose mode of birth highlight the importance of evidence-based practice and the autonomy of physicians, suggesting that patients should not be able to demand a treatment that healthcare providers (HCPs) do not consider appropriate [1, 6] and for which they are legally responsible.

In the context of a marked increase in global caesarean section (CS) rates [12, 13], a central dilemma is whether or not pregnant women should be able to choose a CS without medical indication as mode of birth. CS is a life-saving intervention when clinically indicated. However, the available evidence suggests that carrying out this procedure without clinical indication is associated with increased risks for short and long-term adverse outcomes [14]. Moreover, it has been pointed out that CS upon request diverts resources [1, 15] and places further economic burden in health systems [16].

Decision-making in healthcare settings involves building relations between women and healthcare providers. The relational nature of care has been a long-standing field of study [17]. Recent evidence suggests that HCPs in maternity wards generally welcome women’s increased involvement in the birth process and in decision-making on mode of birth, including CS upon maternal request, especially in high-income countries [7]. Previous research on the topic has focused on the importance of the communication and trust aspects of decision-making, women’s and providers’ values and preferences regarding mode of birth and provision of information and support for women, among others [7, 18–20]. Furthermore, feminist theories have produced an important body of knowledge on the politics of birth [21–23]. However, few empirical studies have focused their attention on how power relations between HCPs and women, and the legal framework in which they develop, shape the way decisions regarding mode of birth are made in healthcare settings [24–26].

### Theoretical framework: How do we understand power relations in maternity care?

In the biomedical model of care, described in Table 1, the traditional patient-doctor relationship is asymmetrical. HCPs are legally and institutionally authorised to make medical decisions [27] and usually decide which treatment options patients are offered [28, 29]. Moreover, as they are considered the legitimate knowledge authority [30], HCPs generally control the terms in which health issues are discussed, which is an important dimension of power in decision-making [31]. This is also the case for the HCP-pregnant woman relationship in maternal health [29]. A study conducted in Australia has shown, for example, how paternalistic practices contribute to women’s lack of control during childbirth [25].

**Table 1:** The biomedical model of care in maternal health

The biomedical model of care in maternal health
• This model was established as a result of birth shifting from a domestic practice led by women to an institutionalized event handled by HCPs in increasingly functional health systems during the 19th and 20th century [22, 34]
• It is characterised by:
• The medicalization of women’s body [35] and their reproductive functions [32, 36, 37], which translates to pregnancy and birth being treated like a disease rather than a physiological process. The pathologization of birth was criticised by feminist theories and the feminist movement as it was considered to legitimise the expansion of medical control at the expense of women’s agency and preferences [22]
• The separation of the biological aspects of reproductive processes from the emotional and subjective ones [38]
• Pregnancy and birth controlled by HCPs in a clinical environment, with a doctor-centred care and decision-making process in maternity wards [39]. This aspect of the biomedical model has been challenged in some high-income countries, in which birth is assisted by different cadres—for example, midwives—and with several concurrent degrees of institutionalization and medicalization [40]

However, power relations established within the biomedical model of pregnancy are not static. Any power dynamics between HCPs and patients involve negotiation and resistance as well as dominance in the medical encounter [17]. In the neoliberal model of medical care, patients in high-income countries have shifted from being considered mere ‘recipients’ of treatment to ‘consumers’ or even ‘reflexive consumers’ of healthcare, suggesting that they more actively participate in the care they receive [32]. In the last few decades, initiatives towards a patient-centred model of care that gives women more control over maternal health have also been proposed [10, 28, 33].

In 2020, Eide and Bærøe, researchers at the University of Bergen, Norway, suggested a concept to structure how decisions regarding mode of birth are reached in maternity wards: *Beneficial power* [29]. It refers to HCPs being able to influence pregnant women’s choice of the best clinical option for them, vaginal birth (VB) or CS, by taking into consideration patients’ own perceptions of needs and desires, especially in the case of CS upon maternal request.

However, this definition of power only works when the doctor-patient relationship is built upon patients’ trusting and valuing HCPs’ opinions and, in turn, HCPs involving patients in the process of decision-making. What happens when this is not the case? Eide and Bærøe address the *opposing autonomous claims* that arise when women ask for a caesarean section without medical indication, which can lead to an *experience of powerlessness* for both providers and patients [29].

In 2004, Law n. 25,929 of Humanized Birth was approved in Argentina. It was regulated in 2015, following the pressure of respectful birth movements to improve the quality of care. This law states the rights of women, families and babies during pregnancy, labour, birth, and postpartum period in healthcare facilities. The law includes women’s right to be treated with respect, to participate in the decision-making process during labour and birth, to be informed about the medical interventions she may receive, to have a companion of choice, to make an informed and free decision regarding where and how to go to labour, among others [41]. There is limited evidence as to how this new law has affected the way in which mode of birth is decided upon in health facilities [42, 43]. In this paper we address the decision-making processes and the interaction between doctors, pregnant women and labour companions when deciding on the mode of birth in a new legal context. Our study reports a qualitative analysis of a formative research about CS determinants carried out in 2018–2019 in Argentina [44]. This is a middle-income country that has seen an increase in rates of CS (34.7% in 2017, according to the National Perinatal Information System), including CS on maternal request [45], during the last few decades. The aim of this qualitative study is to understand the power relations between HCPs, pregnant women, and labour companions in decision-making on mode of birth in hospitals of Argentina from the perspective of HCPs.

## Methods

### Setting

This study was conducted in public maternity wards in Argentina. The health system in this country comprises the public health system, the social security system, and the private health system. The public system is available for anyone who is in Argentinean territory free of charge. The social security system is made up of health insurance coverage managed by trade unions, provincial governments, and other institutions (such as Universities). It is mainly financed by personal contributions from workers and employers, voluntary contributions, co-payments, benefit contributions from the health system, etc. Finally, private companies constitute the private sector.

The majority of births occur in healthcare facilities (99.7%), predominantly in public hospitals (62.5%) [46]. In the public health system, healthcare facilities and providers receive the same payment irrespective of the type of birth, and patients are not charged when receiving care. The national legislation regulates protocols to manage labour and birth. However, there is no specific policy on the use of CS.

### Design

In this qualitative study, we used 26 semi-structured interviews with HCPs. The interviews were carried out during October 2018–August 2019 in a sub-sample of five hospitals as part of a larger mixed-method formative research project in nineteen public hospitals in Argentina, aimed at exploring key informants’ view of the feasibility of implementing non-clinical interventions to reduce caesarean section rates [44]. This formative research was commissioned by the World Health Organization conducted by Centro de Estudios de Estado y Sociedad (CEDES) and Centro Rosarino de Estudios Perinatales (CREP). Results of the mixed-method study have been published elsewhere [47, 48] and have been used to inform the adaptation of non-clinical interventions to optimize the use of CS within the study “Quality decision-making (QUALI-DEC) by women and healthcare providers for appropriate use of caesarean section” [46]. Here we present further in-depth analysis of the qualitative interviews conducted during the formative research.

### Data collection and participants

Five public maternity services in five different jurisdictions of Argentina were selected from the nineteen hospitals participating in the formative research as a sub-sample to carry out in-depth interviews. The criteria to participate in the formative research included using an electronic system to register maternal and perinatal medical records and having more than 1000 births per year. The nineteen facilities were purposively selected from all the eligible hospitals (88 in total) to cover different geographical regions, and for variation in social and economic development as well as health outcomes across the five regions of the country [49]. The sub-sample of five hospitals in which the in-depth interviews were carried out are in the provinces of Santa Fe, Salta, Corrientes, Tucumán and in Buenos Aires City. All participating hospitals provide access to emergency CS and have resources to handle obstetric emergencies [48].

Semi-structured interviews were carried out [50]. The interview guide consisted in 16 open questions and explored the determinants of choice regarding mode of birth, especially focusing on caesarean section births, and key actors’ perceptions regarding the usefulness and feasibility of non-clinical interventions to optimize the use of CS. Two experienced researchers carried out the interviews either in person or virtually, according to the convenience and preference of the interviewees. The interviews had a duration of 30 to 60 min and were recorded.

In each hospital, the participants were purposively selected using heterogeneity sampling regarding professions and positions in the healthcare facilities [44]. Twenty-six HCPs working in five provinces of Argentina were interviewed. Details about jurisdiction, region, and type of provider are presented in Table 2. In hospitals that had midwives in their staff, these were included in the sample. Among obstetricians, the sample included heads of department, specialists working in 24-h shifts, and residents. To recruit participants, researchers from CEDES and CREP contacted a focal person in each hospital, who in turn asked HCPs if they would be interested in participating in the study. Those that accepted to be interviewed were then contacted by a member of the research team.

**Table 2:** Participating maternity hospitals and types of HCPs

Hospital	Jurisdiction	Region	Types of HCPs	Total of interviews per hospital
Hospital 1	Tucumán	Northwest	3 Obstetricians, 2 midwives	5
Hospital 2	Salta	Northwest	2 Obstetricians, 1 obstetric resident, 2 midwives	5
Hospital 3	Santa Fe	Centre	4 Obstetricians, 1 obstetric resident	5
Hospital 4	Corrientes	Litoral	4 Obstetricians, 1 obstetric resident	5
Hospital 5	Ciudad Autónoma de Buenos Aires	Centre	4 Obstetricians, 1 obstetric resident, 1 midwife	6

### Data analysis

We conducted an analysis to explore the power dynamics between women, companions, and HCPs in the mode of birth decision-making process from the perspective of HCPs. The interviews were coded using Nvivo. Reflexive thematic analysis [51, 52] was used to inductively build themes and categories, addressing both semantic and latent content. The first author, MVO, led the coding process with multiple discussions with HMA and CG, who is a member of the research team with strong contextual knowledge who was part of the team conducting the formative research. Consensus throughout the codes was not assumed, so the first author led a process of comparing and contrasting codes together with an insider (CG) and an outsider researcher (HMA). Following several rounds of discussions, we decided how to best develop the themes. Themes were discussed with all co-authors. During the analysis, we compared the five participating hospitals and built crosscutting themes that highlight the commonalities among them regarding decision-making on mode of birth. Therefore, this should be considered a single-case study. The analysis was conducted by an interdisciplinary team made up by researchers with contextual knowledge and expertise in global health.

## Results

Three main themes were developed from the analysis, which are synthesised in Table 3, with their corresponding categories.

**Table 3:** Themes and categories

Overarching theme	HCPs loss of beneficial power in decision-making on mode of birth	
Theme one	HCPs reconceptualize decision-making processes of mode of birth to make women’s voices matter	Temporality
		*Before*: Doctor-centred decision-making
		*Now*: towards a patient-centred decision-making
		Spatiality
		*Changes from inside*: change in HCPs attitudes
		*Changes from outside*: changes in legislation and women’s attitudes
Theme two	HCPs feel powerless against women’s request to choose mode of birth	HCPs frustration when women ask for a CS or birth plan HCPs intent on controlling the process HCPs feel their voice is not heard
Theme three	HCPs struggle to redirect women’s decision regarding mode of birth	HCPs choose not to redirect women’s decision HCPs have difficulty redirecting when there is a window to do so Sometimes it is too late to redirect

### Theme 1: HCPs reconceptualize decision-making processes of mode of birth

Changes in how HCPs conceptualize the decision-making processes regarding mode of birth was emphasized. HCPs claim to be changing their attitudes when deciding between a VB or a CS in the healthcare setting, shifting from a doctor-centred towards a patient-centred model of care. In their view, the former ‘hegemonic’ biomedical model of care was characterised by medical interventionism and doctors’ decision-making. The new model, which is not yet completely instituted, is based in women’s rights and acknowledges patients’ agency to make decisions during the labour and birth process.

The change is usually described using temporal marks like *before* versus *now*, distinguishing a somehow relatively distant past versus current values. Interviewees state that nowadays women’s opinions and wishes are taken into consideration when determining mode of birth in healthcare facilities and that woman are encouraged to take an active role in the process:

*For me, today (…) the power to choose how to give birth lies with the woman, as we have made the slogan of the week of respectful childbirth, to choose how to give birth* (Hospital 1, midwife).

The shift in decision-making conceptualization appears closely related to CS by women’s request. Even though interviewed HCPs agree that CS by maternal request cannot account for the high CS rates in their facilities, it has become an important factor in the doctor-patient interaction regarding mode of birth decision-making.

*One thing that we take into account now and that we did not before is the will of the patient. Before, if a patient told you ‘I want a caesarean section’, we almost did not listen to her, and now it is not that way* (Hospital 3, obstetrician).

The roots of the change were identified both *within* and *without* the health system. Internal changes include HCPs realising that mode of birth is not something that can be imposed by doctors, and that women’s wishes and expectations should be listened to and taken into consideration. As a result, providers are trying to have a less interventionist and paternalist mindset when it comes to labour and birth: ‘We have changed’.

*What I have noticed over the years, what has changed the most is that sometimes the patient has more information, and today the patient is listened to much more. We have all changed as professionals; the patient’s wish for a caesarean section is respected much more than before. Before, she would say, ‘I want a caesarean section’, and one applied a bit of medical paternalism and perhaps led her into a situation… that later we all realised that a patient who does not want to have a vaginal birth is not going to have a vaginal birth* (Hospital 5, obstetrics resident).

On the other hand, changes from outside the health services include the approval and regulation of the Law of Humanised Birth, which is believed to allow choice of mode of birth in clinical settings (and therefore request a CS without medical indication), and changes in the attitudes of pregnant woman and their companions of choice. ‘Patients have changed’, state HCPs, and they underpin this transformation in women’s empowerment, their being more informed regarding their rights and the influence of media.

*I think that women are now quite informed in general, because with all the discussion about the media, the laws, in other words, they are more involved* (Hospital 5, obstetrician).

### Theme 2: HCPs feel powerless against women’s request to choose mode of birth

Even though HCPs claim to have adopted—or be trying to adopt—a new paradigm regarding decision-making, they sometimes feel frustrated when women want to make decisions on mode of birth. Patient-centered decision-making seems to be an acceptable option provided that women choose the option that HCPs, especially doctors, consider appropriate. HCPs state that ‘the decision belongs to the woman’; however, pregnant women and families are many times portrayed as making uninformed ‘wrong’ decisions regarding mode of birth. Fear of litigation in case of adverse neonatal outcomes is presented as a reason to conduct a CS against HCPs’ own judgement, especially when it concerns ‘pushy’ women or companions. In these cases, CS appears to be the only option for some HCPs:

*There are patients who want a caesarean section at all costs, and no matter how much you… you work under a lot of pressure, patients have changed a lot, there is a lot of pressure, sometimes you have patients who pressure you and say “do the caesarean section, if something happens to me it will be your responsibility…”, and even if you don’t want to, at the slightest doubt you are going to have a caesarean section* (Hospital 5, midwife).

In the case of CS without medical indication, HCPs state that they have to accept women’s or companions’ decision regarding mode of birth even if it is not the best clinical option for the patient due to the Law of Humanized Birth. Their ‘voice’ and expertise are no longer taken into account in the matter, as women are protected by law to make “unilateral” decisions. As a result, HCPs feel powerless:

*Because if it [the cesarean section] is by law, it is put “by law” and the pregnant woman’s right is protected by that law (…). By law, we have no say in anything, not as professionals or anything else. The patient has already made the decision and you have to operate, and the problem is over, your speciality is over, just like that* (Hospital 2, obstetrician).

HCPs consider that the wording of the regulation of Law 25,929 of Humanised Birth now allows women to choose mode of birth. This has had consequences in clinical practice, and HCPs report to be performing CSs upon maternal request that are not medically indicated:

*Law 25,929 in its regulation, in the last paragraph of the regulation, those who interpreted it, misinterpreted it… it was different varieties of positions, or that the woman could give birth in any position, and they put mode of birth [vía de determinación], so the woman can choose caesarean section. Today I have already had (…) three caesarean sections by Law 25,929, two nulliparous, which were totally unnecessary, because we didn’t do any trial of labour, or anything, but legally they are allowed* (Hospital 2, head of obstetric service).

HCPs state that some pregnant women also push for a VB when it is not a safe clinical option. In these cases, HCPs do not accept women’s choice, since it might result in an adverse maternal outcome for which they would be legally responsible:

*We have patients with birth plans, in which the patient at all costs wanted a vaginal birth, a vaginal birth, a vaginal birth. However, she had two caesarean sections before, so no, I told her, not here. If you want that, go somewhere else, not here, and she had to accept, because two caesarean sections, the truth is that I am not going to do a vaginal birth* (Hospital 3, head of obstetric service).

The complexities of the decision-making process, as well as the underlying power struggle, makes itself evident when what is questioned it is not the mode of birth that a woman chooses but rather *who* makes the decision and on what basis. HCPs claim that many women make decisions regarding mode of birth without being informed about the risk and benefits of the procedures, even in cases when women state a concrete reason for wanting a CS, for example, not wanting to cope with labour pain.

*Especially in the hospital, you see that we deal with a lower middle class, they are uninformed, and I think that in recent times it is more… they refuse the information that you can give them and they already come with a concept that they do not want pain and they choose a caesarean section, that is, they come with that already, that information in their heads* (Hospital 4, obstetrician).

The issues presented above suggest that HCPs consider *themselves* to be the ones who should be in control of the process of decision-making in the process of labour and birth, both in case of a CS or a VB. This assessment collides with their own accounts of women’s rights and agency.

*But they come with the little piece of paper [the birth plan], they don’t let you touch her, they don’t let you (…) you have to accept it, what do you want me to say? Like the mothers who now don’t want to vaccinate their children, I don’t agree, but… in public health they shouldn’t be allowed to do that* (Hospital 3, head of obstetric service).

It is worth noticing, however, that some midwives in our sample expressed a different point of view than most HCPs (both obstetricians and other midwives) regarding women choosing mode of birth, suggesting, for example, that women choose a CS as part of the empowerment the Law of Humanised Birth aims to enhance:

*Yes, it can be a conditioning factor for a mother who is empowered by the Law on Respectful Childbirth to say, “I decide that I want to have a caesarean section”* (Hospital 1, midwife).

### Theme 3: HCPs struggle to redirect women’s decision regarding mode of birth

HCPs mention three paths in their clinical practice when dealing with CS upon maternal request without medical indication: They sometimes choose not to redirect women’s decision; they sometimes try to change her mind by presenting the risks and benefits of each type of birth, but they struggle to do so; sometimes they consider that it is too late to redirect for legal reasons.

In the first case, HCPs state that the fact that women use the Law of Humanised Birth to ask for a CS, although its original purpose was to protect women against unnecessary interventions. The law is sometimes used as an “excuse” by HCPs to perform CSs, which are viewed as a kind of “easy way out”:

*The patient is also informed about the law, which I think is good, but at the same time (…) the purpose of the law was different, but as it is not clear, they use it the other way round, the purpose of the law was to avoid unnecessary interventions, but as it says that the woman can choose, she can also choose a caesarean section, so in this aspect they [HCPs] take advantage of the situation and say “well, if the woman doesn’t want it [a vaginal birth], she doesn’t want it”, and it ends up being a caesarean section* (Hospital 5, midwife).

Paradoxically, HCPs also emphasize the importance of explaining to women the risk of caesarean section and the process of labour, so that they may change their mind about asking for an unnecessary CS. HCPs mention that communication with women is something they struggle with, and that it is not always possible to get them to change their mind. When HCPs frame this dilemma focusing on changing women’s mind instead of helping them to make an informed decision, it illustrates a wish to prescribe that is part of the paternalistic mindset they claim to have left behind.

*We spend a lot of time talking to patients, we spend more and more time, we criticise those who don’t do it, we even make patients sign a consent form stating that they have been informed of the indication for caesarean section, in order to see if we can lower the rate a little, but what kills us, kills us, is the refusal to try labour* (Hospital 4, obstetrician).

According to HCPs, the reasons why women who demand a CS will not change their minds are manifold. HCPs suggest that women being more informed about their right to choose mode of birth does not mean that they are more aware of the risks and benefits of CS and VB. Not all women access or attend prenatal courses, which results in lack of preparation during pregnancy. Miscommunication between providers and pregnant women on what to expect during the process of labour and misinformation during antenatal care were also mentioned as important factors. Some healthcare professionals also mention that have lost their prestige in the eyes of patients, and therefore their opinions are sometimes not valued.

*It is very difficult to get [pregnant women] to come to the childbirth preparation classes, so sometimes they arrive without knowing what the birth is going to be like, they are often not prepared for the birth, and they also sometimes have a bad opinion of doctors and the [medical] institution. It is something that happens in general, the doctor has lost prestige, and our specialty has lost the admiration of the patients* (Hospital 4, obstetrician).

Finally, sometimes it is too late to redirect women’s request for a CS for legal reasons. Healthcare providers report that due to the Law of Humanised birth, women can ask for a CS when being admitted in the hospital. This decision is written in the medical record of the women, and HCPs cannot attempt to change it:

*That law protects pregnant woman’s right and obviously we have no other option, because the decision has already been made and put in the clinical record, so we don’t…. We are not involved in any kind of decision; the decision has already been made by the patient* (Hospital 2, obstetric resident).

## Discussion

Choice of mode of birth is a complex process influenced by a vast array of issues. Our analysis raised the importance of power struggles between HCPs in decision making on the mode of birth. While providers claim to believe that they have changed previous paternalistic practices, allowing pregnant women to become an active actor in decision-making, they feel frustrated when they are not able to influence women’s choice of mode of birth. We have conceptualized this as loss of *beneficial power* [29]. Paradoxically, providers sometimes use women’s request for a CS as an excuse to perform a surgical birth, since it appears to be “easier” for them. This suggests that the interviewees are not against performing CS per se, but rather that they want to retain control over decision-making.

The loss of *beneficial power* is also experienced by HCPs when women or their companions question their knowledge and prestige. In the traditional biomedical model of care, doctors were the ones to define the terms and boundaries of the discussions with patients, given their legitimised expertise [17, 28, 30]. This provided them with more control over the decision-making process. Interviewed HCPs, especially medical doctors, perceive their authority as being challenged and have trouble making women and family “listen” to their medical advice. A previous qualitative study has similarly suggested that HCPs find shared-decision making to be hindered when patients request medical practices that do not align with HCPs experiences or knowledge [26].

In the last few decades, CS at women’s request has been a key aspect of the controversy regarding decision-making on mode of birth. A recent mixed-methods study conducted in Argentina on the views of obstetricians, midwives and residents in public healthcare facilities showed that there was no agreement among professional groups on whether CS upon request is a determinant of the CS increase in this country [48]. Although there is no updated information about the proportion of CS due to maternal request, our study suggests that it appears to play an important role in the doctor-patient interaction regarding mode of birth. The Law of Humanised Birth has a central place in HCPs accounts regarding decision-making on mode of birth. HCPs believe that, because of the way it is written, the law has in practice allowed for choice on mode of birth. This, in turn, appears to have had a considerable impact on power relations among HCPs, pregnant women and companions and the way decisions are made in the clinical settings. More research is needed to understand how the Law of Humanised Birth is currently interpreted in maternity wards regarding choice of mode of birth.

HCPs feel powerless upon women being legally able to ask for a CS without medical indication. They mention fear of litigation as a significant reason for agreeing to perform a CS upon request, especially when pregnant women or family members put pressure on them. This has also been pointed out by previous literature as an important determinant of CS increase [53]. A recent study in Mexico also showed that HCPs felt powerless and frustrated upon women and families asking for a CS, especially when faced with difficult or aggressive patients [24].

Women’s lack of preparation during pregnancy and misinformation during antenatal care regarding what to expect during the labour process were suggested as important reasons why pregnant women refuse to try a VB, especially based on not wanting to cope with labour pain. This is in line with previous literature, which has shown that fear of pain during labour is a reason for women to prefer a CS [9, 54–58]. Recent quantitative studies on women’s preferences on mode of birth in Argentina and Brazil indicated that avoiding pain was one of the main reasons put forward by women preferring a CS to a VB [47, 59]. An important contextual factor to take into consideration is that only one out of the five participating hospitals provides epidural analgesia during labour as a routine practice.

HCPs also mention misinformation about the risks and benefits of each mode of birth as a contributing factor for CS upon maternal request. The aforementioned study from Argentina showed that the main reason for women preferring a CS was that they perceived it as a safer option than VB [47], which is in line with research conducted in other countries [57, 60–63] but contradicts the available evidence on the risks and benefits of each mode of birth [14, 64]. According to the interviewees, women are also influenced by media, which has been identified as having a significant role in agenda setting for fashionable trends regarding mode of birth [65].

Finally, women look for meaningful communication and interaction with their HCPs when deciding on mode of birth [66, 67]. Our study showed that miscommunication between pregnant women and HCPs is a factor that plays a role in the power dynamics regarding mode of birth decision-making. HCPs emphasize the importance of explaining the risks and benefits of each type of birth to women, yet they report that they do not always find it easy to interact with them. More research is needed to understand why HCPs in Argentina find it difficult to establish a fruitful conversation with pregnant and labouring women in healthcare facilities.

The literature on the topic suggests that a *shared process of decision-making* [29] may be the best way to disentangle the complex issue of choosing mode of birth [9, 29, 55, 65, 68]. Evidence has suggested that women being in control and able to choose during the process enhances the birth experience [69–71]. However, certain conditions must be met for the decision to be taken together by HCPs and pregnant women. Taking into account women’s opinions, values and desires, as well as respecting their autonomy and agency has been suggested as paramount [29]. Reliable and updated information provided to women in healthcare facilities that will allow them to weigh the risks and benefits of each mode of birth has been considered essential [57, 66, 72]. Moreover, meaningful communication and interaction with HCPs has been shown to be an important factor for women when making a decision on mode of birth, and therefore should be consider a fundamental aspect of shared decision-making [19, 66, 73]. Finally, provision of emotional support before and during labour has also been shown to help women in the decision-making process [66, 67].

## Limitations

This qualitative study used the audio recording and qualitative interview transcripts of a formative research conducted in Argentina [44], collected with a different aim and using a guide that was not exclusively tailored to our research questions. Moreover, data saturation was decided in line with the purpose of the formative research. Even though data was not collected with the purpose of looking at power relations, this issue was addressed in several parts of the interviews. For example, during this data-driven analysis, we were able to compare how power was expressed at different stages: antenatal care, admission, labour, and birth. Moreover, it was a topic tackled by different types of HCPs (OBGYN specialists, midwives and residents) in all participating hospitals. It should be mentioned that only three of the five included hospitals had midwives in their staff, hindering the comparison between different cadres per hospital.

We report on very specific power relations between HCPs and pregnant women in five public maternity wards in Argentina in the context of a new legal scenario regarding mode of birth. Transferability to other contexts includes public hospitals in this country where statistics on CS are available, as well as other contexts with similar legislation or in which the practice of CS upon medical request has been a concerning issue. We also consider the results of this study to potentially be of use to researchers, decision-makers, policy makers and HCPs in settings that are trying to shift from a doctor-centered towards a patient-centered model of maternity care.

## Conclusion

Decision-making on mode of birth remains an intricate debate that should not only look into medical research findings, women’s and providers’ autonomy, ethics, law and media, but also into clinical practice and the interaction between HCPs, women and companions. This study suggests that power relations within the healthcare setting and the legal framework in which these relations take place are important dimensions to take into account in the mode of birth controversy. Our analysis highlights the complexity of the HCP-patient interaction in the context of a Law of Humanised Birth that, in practice, is understood as allowing women to choose mode of birth. Even though HCPs claim to be changing their biomedical mindset, welcoming women to be an active part of the decision-making processes in labour and birth, they feel frustrated and powerless when women make autonomous decisions regarding mode of birth, especially in the case of CS without medical indication. HCPs perceive themselves to be losing *beneficial power* and prestige in the eyes of patients, and that fruitful communication on risks and benefits of each mode of birth is not always possible in the mode of birth decision-making process. Furthermore, HCPs perform more and more CS without medical indication when it is convenient for them, which suggests that paternalistic practices and beliefs are still in place.

## Data Availability

Data are available on reasonable request.

## Abbreviations

CS: Caesarean section
HCP: Healthcare provider
OBGYN: Obstetrician/gynaecologist
VB: Vaginal birth
